# Serum Cholinesterase Inhibition in Relation to Paraoxonase-1 (PON1) Status among Organophosphate-Exposed Agricultural Pesticide Handlers

**DOI:** 10.1289/ehp.0900682

**Published:** 2009-06-09

**Authors:** Jonathan N. Hofmann, Matthew C. Keifer, Clement E. Furlong, Anneclaire J. De Roos, Federico M. Farin, Richard A. Fenske, Gerald van Belle, Harvey Checkoway

**Affiliations:** 1 Department of Epidemiology; 2 Department of Environmental and Occupational Health Sciences; 3 Department of Medicine, Division of Medical Genetics and; 4 Department of Genome Sciences, University of Washington, Seattle, Washington, USA; 5 Fred Hutchinson Cancer Research Center, Seattle, Washington, USA; 6 Department of Biostatistics, University of Washington, Seattle, Washington, USA

**Keywords:** agriculture, cholinesterase, farmworkers, gene-environment interaction, organophosphates, paraoxonase, pesticides

## Abstract

**Background:**

Animal studies have demonstrated that low paraoxonase-1 (PON1) status (i.e., low catalytic efficiency and/or low plasma PON1 activity) is associated with neurotoxic effects after exposure to several organophosphate (OP) insecticides. However, few human studies have investigated associations between PON1 status and intermediate end points, such as serum cholinesterase [butyrylcholinesterase (BuChE)] inhibition, among OP-exposed individuals.

**Objectives:**

We evaluated the relation between plasma PON1 status and BuChE inhibition among OP-exposed agricultural pesticide handlers.

**Methods:**

Agricultural pesticide handlers in Washington State were recruited during the 2006 and 2007 spray seasons when they were seen for follow-up ChE testing by collaborating medical providers as part of a statewide monitoring program. Blood samples were collected from 163 participants and tested for PON1 status based on plasma PON1 activity [arylesterase (AREase)] and *PON1* Q192R genotype. We evaluated percent change in BuChE activity from baseline level in relation to PON1 status.

**Results:**

We observed significantly greater BuChE inhibition among QQ homozygotes relative to RR homozygotes (*p* = 0.036). Lower levels of plasma PON1 activity were significantly associated with greater BuChE inhibition (*p* = 0.004). These associations remained after adjustment for year, days since baseline test, age, and OP exposure in the last 30 days.

**Conclusions:**

We found that both low PON1 catalytic efficiency (i.e., the Q192 alloform) and low plasma PON1 activity were associated with BuChE inhibition among OP-exposed agricultural pesticide handlers. Corroborative findings from future studies with prospective collection of blood samples for PON1 testing, more sensitive markers of OP-related effects, and larger sample sizes are needed.

Organophosphate (OP) and *N*-methyl-carbamate (CB) insecticides inhibit cholinesterase (ChE) enzyme activity, including both serum ChE [butyrylcholinesterase (BuChE)] and erythrocyte ChE [acetylcholinesterase (AChE)]. OP/CB insecticides are widely used in agriculture in the United States and abroad. Washington State is the nation’s leading producer of apples and several other tree fruits, and OP/CBs are applied in many orchards in eastern Washington. In 2004, the Washington State Department of Labor and Industries (WADLI) initiated a ChE monitoring program for agricultural pesticide handlers who are exposed to toxicity category I or II OP/CBs (WADLI 2004). In this program, pesticide handlers (agricultural workers who apply pesticides or are otherwise involved in the pesticide application process) are tested for AChE and BuChE activities annually before the OP/CB spray season (i.e., at baseline), and during the spray season if they handle OP/CBs for ≥ 30 hr in a 30-day period. Handlers who are exposed only to CBs (not OPs) are not required to come in for follow-up ChE tests. Consequently, the vast majority of handlers in this monitoring program had been recently exposed to OPs over a period of several days to weeks at the time of their follow-up ChE test. Follow-up test results that show evidence of a decrease in AChE or BuChE activity from baseline levels can lead to work practice evaluations or removal from continued OP/CB exposure (with wage protection), depending on the degree of ChE inhibition observed.

Inhibition of AChE activity in the central and peripheral nervous systems is considered to be the main mechanism of OP/CB toxicity (Ecobichon 2001). In addition to being found in the nervous system, AChE is present on red blood cell membranes. BuChE is synthesized in the liver and is present in serum. Both AChE and BuChE activity can be measured in blood specimens as surrogates for neuronal AChE activity. Although AChE activity in blood is thought to more closely approximate neuronal AChE activity than does BuChE (Lotti 2001), both are considered valid markers of OP/CB-related biological effects by the U.S. Environmental Protection Agency (U.S. EPA 2000).

The health effects of acute OP/CB poisonings have been well characterized (Ecobichon 2001), and a growing body of evidence suggests that various health end points may be associated with chronic occupational OP/CB exposure. Previous studies have shown that OP exposure may be associated with deficits in neurobehavioral performance (Rothlein et al. 2006), chronic neurologic effects (Kamel and Hoppin 2004; Kamel et al. 2005), and several types of cancer (Alavanja et al. 2004).

It is possible that some individuals may be especially susceptible to health effects related to OP/CB exposure. High-density lipoprotein–associated paraoxonase-1 (PON1) is thought to be one important determinant of an individual’s sensitivity to some OP insecticides, based primarily on evidence from studies in animal models (Cole et al. 2005; Li et al. 2000; Shih et al. 1998). PON1 hydrolyzes the highly toxic oxon forms of several widely used OPs, including chlorpyrifos and diazinon. PON1 does not hydrolyze all OPs (e.g., azinphos-methyl does not appear to be a PON1 substrate), and the role of PON1 in metabolism of CBs has not been investigated. Studies in transgenic mice have clearly demonstrated that low plasma PON1 activity is associated with greater brain AChE inhibition after exposure to chlorpyrifos oxon and diazoxon (the oxon forms of chlorpyrifos and diazinon) (Li et al. 2000). Also, a Q/R polymorphism at position 192 in the *PON1* coding region affects the catalytic efficiency of the enzyme for chlorpyrifos oxon metabolism. In a study by Cole et al. (2005) of mice expressing equivalent levels of the different alloforms of humanized PON1, greater brain AChE inhibition was observed among mice with the Q192 alloform relative to the R192 alloform after chlorpyrifos oxon exposure.

Several important points should be considered regarding PON1-mediated sensitivity to OP exposure: *a*) PON1 status is most relevant for protection against direct exposure to the oxon forms of OP insecticides (Li et al. 2000; Shih et al. 1998); *b*) most, if not all, OP exposures may include oxon residues (California Environmental Protection Agency 1998; Yuknavage et al. 1997); and *c*) the safety studies for OPs such as chlorpyrifos were carried out with the highly pure parent compound (Nolan et al. 1984).

Despite convincing evidence in animal models, few epidemiologic studies have evaluated PON1 status as a determinant of OP sensitivity. We recruited agricultural pesticide handlers from the Washington State ChE monitoring program for a study to test the hypothesis that PON1 status affects an individual’s risk of BuChE inhibition and is thus a biomarker of susceptibility.

## Methods

Agricultural pesticide handlers in the statewide ChE monitoring program were recruited for this study from two collaborating clinics in eastern Washington during the 2006 and 2007 spray seasons (April–July). Blood samples for determination of PON1 status and self-reported OP/CB exposure information were collected at the time of follow-up ChE testing during the spray season. A total of 163 participants with nonhemolyzed blood specimens for PON1 testing and both baseline and follow-up ChE tests were included in this analysis. This sample represents 54% of the 304 handlers who were invited to participate. Study participants were similar to all handlers in the statewide ChE monitoring program in terms of age, race/ethnicity, and sex [Scientific Advisory Committee for Cholinesterase Monitoring (SAC) 2006]. We excluded one individual from this analysis because of hemolysis in the plasma sample for PON1 testing. Some of the handlers enrolled in this study participated in both years (*n* = 25). For these individuals, the record with greater BuChE inhibition was included in the data set, and the other record was excluded (i.e., oversampling records with BuChE inhibition). Analyses were also repeated with random selection of a single record for these individuals who participated in both years.

In addition to collection of blood specimens for PON1 testing, participants were asked to complete a computer-based survey regarding OP/CB exposure in the preceding 30 days and demographic characteristics. Surveys were completed by 141 of the participants included in this analysis. Another 11 participants had partially complete surveys; nonmissing data from incomplete surveys were used where applicable.

This study was conducted in compliance with all applicable U.S. requirements for research involving human subjects, and all study procedures were approved by the institutional review board at the University of Washington. Study participants provided written informed consent before participating in this study.

### Determination of PON1 status

Handlers’ PON1 status was characterized based on genotype at position 192 in the *PON1* coding region and level of plasma PON1 activity. Genotype at position 192 was determined using a previously established TaqMan-based genotyping assay (Applied Biosystems Inc., Foster City, CA) with genomic DNA extracted from EDTA-treated whole-blood specimens (Searles Nielsen et al. 2005). The genotype distribution was not significantly different from Hardy–Weinberg equilibrium expectations (*p* > 0.9).

The level of plasma PON1 activity was characterized by measuring arylesterase (AREase) activity in lithium heparin–treated plasma samples; AREase activity is considered to be a good surrogate for PON1 concentration in plasma (Furlong et al. 1993, 2006). Phenylacetate was used as the substrate in these assays, and hydrolysis rates were reported in units per milliliter.

### BuChE measurements

All study participants were tested for annual baseline BuChE activity (generally a few weeks before the OP/CB spray season) and at follow-up during the spray season as part of the statewide monitoring program. ChE test results were obtained from collaborating health care providers. Methods for collection and processing of blood specimens for ChE testing have been described previously (WADLI 2006). Because of an administrative change in the state program, BuChE tests were performed by two different laboratories: the Washington State Public Health Laboratory (PHL; Shoreline, WA) in 2006 and Pathology Associates Medical Laboratories (PAML; Spokane, WA) in 2007. Baseline and follow-up BuChE tests were performed by the same laboratory within each year. Both laboratories measured BuChE activity using the Ellman method with reagents from Roche Diagnostics (Ellman et al. 1961). However, different instruments were used by each lab: The PHL used an automated Dade Dimension AR system, and PAML used an Olympus AU5421/AU2700 system. Although absolute levels of BuChE activity differed between the two labs, both had high internal precision for BuChE measurements. Coefficients of variation (CVs) were 2.5% for PHL in 2006 and 2.6% for PAML in 2007 (WADLI 2007).

In this study, we focused on BuChE inhibition for several reasons. First, BuChE is more sensitive than is AChE to inhibition by many OPs, including chlorpyrifos, which was the most widely used OP among study participants (Amitai et al. 1998; Lotti 2001). Second, there was little evidence of AChE inhibition among study participants or among all handlers in the statewide monitoring program. Among the 472 handlers in the state monitoring program who had baseline tests and at least one follow-up test in 2006, mean AChE inhibition was 1.8%, and only two handlers had “AChE depression” at the work practice evaluation threshold of > 20% inhibition (SAC 2006). Finally, high variability in AChE measurements was observed in analyses of data from the state monitoring program in 2007 (CV, 16.7%) (WADLI 2007); this would likely have obscured any associations between PON1 status and AChE inhibition in our study.

### Statistical analysis

The primary aim of this study was to test the hypothesis that low PON1 status is a determinant of BuChE inhibition among OP-exposed pesticide handlers. For these analyses, PON1 status was characterized in terms of both observed Q192R genotype and level of plasma PON1 activity (AREase activity). The primary end point was percent change in BuChE activity comparing levels at follow-up during the OP/CB spray season with preseason baseline levels. We considered the degree of change in BuChE to be the most biologically relevant end point because of wide interindividual variability in baseline BuChE activity (Cocker et al. 2002; Sidell and Kaminskis 1975). We also repeated analyses using follow-up BuChE activity as the outcome variable, with baseline BuChE activity included as a covariate in the model.

We performed several different analyses to characterize the relation between PON1 status and BuChE inhibition. First, we evaluated mean BuChE inhibition from baseline after stratifying by both Q192R genotype and tertiles of plasma PON1 activity. We compared the degree of BuChE inhibition within each stratum with the reference category of individuals who were hypothesized to have the lowest risk (i.e., individuals with RR genotype and high plasma PON1 activity). Comparisons were based on linear regression with robust standard error estimates. We also performed linear regression with plasma PON1 activity modeled as a continuous predictor of BuChE inhibition after stratification by Q192R genotype.

We performed linear regression analyses to evaluate BuChE inhibition by Q192R genotype and level of plasma PON1 activity with and without adjustment for covariates. Analyses were also repeated after stratifying by year of participation. Adjusted models included both Q192R genotype and plasma PON1 activity, as well as year of participation, days since baseline ChE test, age in years, and cumulative OP/CB exposure score for the preceding 30 days. Age was reported by participants as part of the survey; for some participants with missing self-reported age (*n* = 19), we used birth dates from administrative records that were collected as part of the ChE monitoring program. Cumulative OP/CB exposure within the preceding 30 days was estimated quantitatively based on survey responses regarding OP/CB use, work activities, and personal protective equipment (PPE) use (Hofmann JN, unpublished observations). Briefly, we calculated a score for OP/CB toxicity based on data from U.S. EPA cumulative risk assessments, and scores for work activities and PPE use based on algorithms developed as part of the Agricultural Health Study (Dosemeci et al. 2002; U.S. EPA 2005, 2006). Variables for OP/CB toxicity, work activities, and PPE use were transformed into *z*scores, and cumulative-OP/CB exposure was estimated by adding together the *z*-score values. Because plasma PON1 activity appeared to decrease slightly with increasing cumulative OP/CB exposure score, we treated OP/CB exposure score as a potential confounder, including it as a covariate in all adjusted analyses.

In addition to linear regression analyses, we also used unconditional logistic regression to evaluate risk of “BuChE depression,” defined as > 20% BuChE inhibition from baseline activity level, by Q192R genotype and tertiles of plasma PON1 activity. We adjusted for the following covariates in this analysis: year of participation (2006 (2007), time since baseline ChE test (≤ 60 days, 61–90 days, > 90 days), age (18–24, 25–34, 35–49, ≥ 50 years), and cumulative OP/CB exposure score (< −0.85, −0.85 to 0.45, > 0.45).

We performed several exploratory analyses in this study. Linear regression analyses were repeated after restricting to chlorpyrifos-exposed handlers, and after excluding handlers who reported only using CB insecticides. We also conducted linear regression analyses for BuChE inhibition in relation to Q192R genotype and level of plasma PON1 activity after stratifying by tertiles of OP/CB exposure to evaluate whether the relations between PON1 status and BuChE inhibition differed by degree of OP/CB exposure.

All statistical analyses were performed using Intercooled Stata, version 9.2 (StataCorp, College Station, TX). Findings were considered statistically significant if *p*-values were < 0.05.

## Results

Characteristics of study participants are reported in Table 1. All of the participants in this study were male, and all but one with reported ethnicity were Hispanic/Latino. Approximately 60% of study participants were < 35 years of age. In terms of reported OP/CB use in the preceding 30 days, chlorpyrifos was the most widely used compound (67%), followed by carbaryl (30%) and azinphosmethyl (14%). Approximately two-thirds of the study participants handled OP/ CB insecticides within the week before their follow-up ChE test (64%).

### PON1 status and BuChE inhibition

Overall, participants in this study had significantly lower BuChE activity at the time of follow-up during the OP/CB spray season relative to baseline (i.e., preseason) levels (*n* = 163; *p* < 0.001, paired *t*-test). Mean BuChE inhibition from baseline activity was 5.5% (range, −37.0% to 18.4%); this is consistent with the degree of BuChE inhibition observed among all handlers in the statewide ChE monitoring program (SAC 2006). Relative to individuals with RR genotype and high plasma PON1 activity (i.e., hypothesized lowest risk individuals), those with either QQ genotype or low plasma PON1 activity had significantly greater BuChE inhibition (Table 2). Handlers with both QQ genotype and low plasma PON1 activity (i.e., hypothesized highest risk individuals) had the greatest degree of BuChE inhibition of any group. A test for trend in BuChE inhibition by number of Q alleles and plasma PON1 activity levels was highly significant (*n* = 163, *p* = 0.002). Differences in AREase category frequencies within each Q192R genotype were not statistically significant (*p* = 0.118, chi-square test).

**Figure 1:**
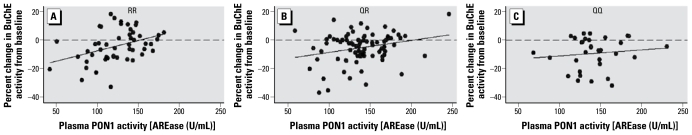
BuChE inhibition in relation to level of plasma PON1 activity, stratified by *PON1* Q192R genotype (*n* = 163). Coefficients based on robust linear regression were, for (*A*) RR, β = 0.145, *p* = 0.001; for (*B*) QR, β = 0.083, *p* = 0.068; and for (*C*) QQ, β = 0.044, *p* = 0.341.

Figure 1 shows the linear trend between BuChE inhibition and level of plasma PON1 activity after stratification by Q192R genotype (*n* = 163). Based on this analysis, Q192R genotype appears to modify the relation between plasma PON1 activity and BuChE inhibition. A highly significant association between lower plasma PON1 activity and greater BuChE inhibition was observed among RR individuals (*n* = 50, *p* = 0.001). This suggests that even individuals with the high catalytic efficiency genotype (192R homozygotes) for chlorpyrifos oxon hydrolysis may still be at risk of BuChE inhibition if they have low plasma PON1 activity. There was a borderline significant relationship between plasma PON1 activity and BuChE inhibition among QR individuals (*n* = 81, *p* = 0.068), and we observed no association among QQ individuals (*n* = 32, *p* = 0.341). However, handlers with QQ genotype had significantly greater BuChE inhibition than did RR individuals (*p* = 0.036), which suggests that low catalytic efficiency of chlorpyrifos oxon hydrolysis may be a determinant of BuChE inhibition regardless of the level of plasma PON1 activity.

We also evaluated the degree of BuChE inhibition in relation to Q192R genotype and plasma PON1 activity after adjusting for covariates (Table 3). As in the unadjusted analyses, QQ homozygotes had significantly greater BuChE inhibition than did RR homozygotes after adjustment (*n* = 110, *p* = 0.03). We also observed a significant trend toward greater BuChE inhibition by number of Q alleles (*p* = 0.028). We observed a borderline significant association between level of plasma PON1 activity (modeled as a continuous predictor) and BuChE inhibition in the adjusted analysis (*p* = 0.053). Analyses stratified by year of participation indicated that the associations between Q192R genotype and BuChE inhibition within each year were similar to the results of the main adjusted analysis. Although we observed greater BuChE inhibition with decreasing AREase activity in both years, this association was stronger in 2006 than in 2007 (β = 0.073 and 0.039, respectively). However, a test for interaction by year was not statistically significant (*p* = 0.529). Results of adjusted analyses evaluating follow-up BuChE activity were generally consistent with the results for BuChE inhibition (*n* = 110, *p* = 0.034 for QQ vs. RR genotype, and *p* = 0.066 for plasma PON1 activity). When we restricted the analyses to chlorpyrifos-exposed handlers, associations between BuChE inhibition and Q192R genotype and plasma PON1 activity after covariate adjustment were similar in magnitude but no longer statistically significant (*n* = 69, *p* = 0.119 for QQ vs. RR genotype, and *p* = 0.092 for plasma PON1 activity). Similarly, results were essentially unchanged after excluding 21 handlers who reported using only CB insecticides (*n* = 89, *p* = 0.021 for QQ vs. RR genotype, and *p* = 0.094 for plasma PON1 activity). When analyses were repeated after a single record from handlers who participated in both years was selected at random (rather than sampling based on BuChE inhibition), similar results were obtained (data not shown).

As shown in Table 4, after adjustment for covariates, individuals with the QQ genotype were approximately 10 times as likely as RR individuals to experience BuChE depression (i.e., > 20% BuChE inhibition; *n* = 110, *p* = 0.036). Relative to individuals with high plasma PON1 activity, those with low plasma PON1 activity were three times as likely to have BuChE depression; this association was not statistically significant. However, when level of plasma PON1 activity was modeled as a continuous predictor, a statistically significant decrease in odds of BuChE depression was observed with increasing plasma PON1 activity (*p* = 0.042). There were relatively few cases of BuChE depression in the study population (*n* = 15 for the adjusted analysis). Consequently, the confidence intervals for these odds ratios are fairly wide, and results should be interpreted with caution.

We also performed several analyses to evaluate whether effect estimates for the association between PON1 status and BuChE inhibition differed by degree of OP/CB exposure. After stratifying by tertiles of OP/CB exposure score, we found that the magnitude of the association between QQ genotype (vs. RR genotype) and BuChE inhibition increased with greater OP/CB exposure (β = −3.38, −4.77, and −9.18 for low, medium, and high OP/CB exposure categories, respectively). We observed no such trend in the relationship between plasma PON1 activity and BuChE inhibition after stratification by OP/CB exposure.

## Discussion

The relation between PON1 status and OP toxicity has been well characterized in animal models (Cole et al. 2005; Li et al. 2000; Shih et al. 1998). However, relatively few epidemiologic studies have investigated PON1 status as a biomarker of susceptibility to OP-related effects. We found that both the *PON1* Q192 alloform and low plasma PON1 activity were determinants of BuChE inhibition among OP-exposed agricultural pesticide handlers. These findings are consistent with expectations based on results from animal studies (Cole et al. 2005; Costa et al. 2005a; Li et al. 2000).

Several previous studies have evaluated PON1-related sensitivity among OP-exposed individuals. A case–control study by Mackness et al. (2003) of self-reported chronic ill health among sheep dippers exposed primarily to diazinon indicated a 2.5-fold higher risk among farmers in the lowest quintile of diazoxonase activity compared with the farmers in the highest quintile. However, that study did not take into consideration the fact that the R alloform of PON1 has somewhat better catalytic efficiency for diazoxon than the Q alloform both *in vitro* and *in vivo* (Li et al. 2000; Richter et al. 2009). Another study by Lee et al. (2003) evaluated PON1 Q192R genotype among OP-exposed fruit farm workers in South Africa. Relative to RR individuals, those who were either QR heterozygotes or QQ homozygotes were almost three times as likely to report multiple (≥ 2) symptoms of chronic OP toxicity (e.g., abdominal pain, headache, gait disturbance, and limb numbness among other symptoms). In a case–control study of acute OP intoxication, Sozmen et al. (2002) found that cases had a significantly higher frequency of the PON1 Q192 alloform and lower paraoxonase (POase) activity than did controls. They also found that POase activity was lower among cases with low BuChE activity upon hospital admission relative to cases with higher BuChE activity, suggesting a protective effect of PON1 against BuChE inhibition.

The association between low plasma PON1 activity and BuChE inhibition that we observed in this study is consistent with the results of some (Mackness et al. 2003; Sozmen et al. 2002) but not all (Perez-Herrera et al. 2008) studies that evaluated adverse health effects in relation to PON1 enzyme activity, even though different substrates to evaluate PON1 enzyme activity in several of these studies. The association between the Q192 alloform and greater BuChE inhibition in this study is also consistent with findings from studies that evaluated symptoms of chronic pesticide toxicity (Lee et al. 2003) and acute OP poisonings (Sozmen et al. 2002). However, other studies have reported associations between the R192 alloform and increased risk of chronic ill health (Mackness et al. 2003) and adverse effects on semen quality and sperm DNA integrity (Perez-Herrera et al. 2008). Inconsistencies in findings between studies may be attributable to exposure to different OP/CB compounds with varied toxic effects (Mackness et al. 2003; Perez-Herrera et al. 2008) or evaluation of other health end points that are unrelated to ChE inhibition (Perez-Herrera et al. 2008).

This study had noteworthy strengths. By recruiting participants from the recently implemented Washington State ChE monitoring program, we were able to establish a cohort of agricultural pesticide handlers with confirmed recent OP exposure. Because pesticide handlers (i.e., mixer/loader/applicators) are considered to be more highly exposed to pesticides than agricultural workers who perform other activities, this population is especially well suited for evaluating PON1-mediated susceptibility to OP exposure. Previous studies of PON1-related susceptibility among individuals with occupational OP exposure have relied on self-reported health outcomes such as chronic ill health (Mackness et al. 2003) or symptoms of chronic toxicity (Lee et al. 2003). In the present study, we were able to use BuChE inhibition as a quantitative biomarker of OP-related effects as our primary outcome. Finally, some previous studies have relied exclusively on PON1 genotype. However, it is also important to consider the level of plasma PON1 activity, which can affect an individual’s ability to metabolize OPs at physiologically relevant rates independently of the Q192R polymorphism. For some (but not all) OPs that are metabolized by PON1, the Q192R polymorphism is also important because it affects the catalytic efficiency of OP hydrolysis. For example, both the Q192R genotype and the level of plasma PON1 activity affect chlorpyrifos oxon metabolism (Cole et al. 2005). Determination of both PON1 Q192R genotype and level of plasma PON1 activity in this study allowed for a better characterization of overall PON1 status than available with genotype alone (Richter and Furlong 1999).

This study had several limitations. There is the possibility of uncontrolled confounding leading to inaccurate assignment of PON1 status because PON1 activity may be modified to some extent by certain medications (e.g., statins), dietary habits (e.g., vitamin C and E intake), and environmental exposures (e.g., smoking) (Costa et al. 2005b; Durrington et al. 2002; Jarvik et al. 2002). However, very few participants in this study reported using cholesterol-lowering medications (2.4%), and risk estimates were essentially unchanged after controlling for smoking status (data not shown). Moreover, most previous studies suggest that plasma PON1 activity levels tend to be relatively stable over time and are regulated mostly by genetic factors, particularly the C108T promoter polymorphism (Brophy et al. 2001a, 2001b; Durrington et al. 2002; Ferre et al. 2003; Furlong et al. 2000; Jarvik et al. 2002; Leviev and James 2000; Suehiro et al. 2000; Zech and Zurcher 1974). Another limitation is the absence of PON1 and BuChE measurement data after the OP/CB spray season, which would help clarify the transient or persistent nature of effects. We are not aware of other similar studies that have included measurements before, during, and after the spraying season.

Absolute measurements of BuChE activity differed in 2006 and 2007 because of an administrative change in the laboratory conducting ChE testing for the statewide monitoring program. However, within each year baseline and follow-up ChE tests were performed by the same laboratory, so we were able to use the degree of BuChE inhibition from baseline levels as the main end point even though absolute BuChE measurements differed by year. Moreover, we included year of participation as a covariate in all adjusted models to control for differences by laboratory. Other newly developed biomarkers of OP exposure could potentially address concerns about BuChE activity measurements and may be studied in the future (Quistad et al. 2005; Richards et al. 1999).

The relatively low participation rate in our study (54%) may be another limitation. However, study participants were similar to all participants in the statewide ChE monitoring program in terms of demographic characteristics and the degree of BuChE inhibition observed (SAC 2006), which suggests that our sample is likely to be representative of agricultural pesticide handlers participating in the ChE monitoring program in Washington State. Among study participants, missing data for some variables were also a concern. Approximately one-third of the records in the data set were excluded from adjusted analyses because of missing data. Most of the excluded records were missing data for the OP/CB exposure score variable because some participants provided blood samples for PON1 testing but did not complete the survey or were unable to identify specific OP/ CB insecticides used in the preceding 30 days. However, there did not appear to be any substantial differences in terms of PON1 status or BuChE inhibition between the records that were included in the adjusted analyses and records that were excluded.

Compared with RR individuals, relatively few QQ individuals had low plasma PON1 activity. It is possible that these differences in plasma PON1 activity levels by Q192R genotype may be attributable to the healthy worker survivor effect (e.g., QQ individuals with low PON1 activity might be more susceptible to pesticide-related illness and drop out of the work force before follow-up). However, given the relatively low frequency of acute pesticide-related illnesses in this population (Washington State Department of Health 2008), such differences in plasma PON1 activity by Q192R genotype could also be explained by chance. Even if the healthy worker survivor effect is present, we would expect this type of bias to attenuate any observed associations. Consequently, the associations observed in this study can be interpreted as conservative risk estimates.

Finally, although we had a larger sample size than most previous studies of PON1-mediated OP sensitivity (Lee et al. 2003; Perez-Herrera et al. 2008; Sozmen et al. 2002), this study had limited power to detect associations in the logistic regression analyses of BuChE depression and when evaluating interaction between OP/CB exposure score and PON1 status in relation to BuChE inhibition.

## Conclusions

In this study we found that both low PON1 catalytic efficiency (i.e., the Q192 alloform for chlorpyrifos oxon hydrolysis) and low plasma PON1 activity were associated with BuChE inhibition among OP-exposed agricultural pesticide handlers. Regulatory risk assessments should take differences in PON1-related sensitivity to OP insecticides into consideration when characterizing interindividual variability in risk related to OP exposure. At some point in the future, biologic monitoring for PON1 status among pesticide handlers may be warranted to identify individuals who are at particularly high risk of OP-related health effects. However, issues of test validity as well as the ethical and legal aspects of genetic testing in the workplace will need to be addressed before such a program could be implemented (Battuello et al. 2004).

Findings from this study need to be confirmed in future studies, which would benefit from *a*) collection of blood specimens for determination of plasma PON1 activity before, during, and after exposure periods; *b*) evaluation of both AChE and BuChE inhibition as end points; *c*) other newly developed biomarkers of OP exposure; and *d*) a larger sample size to assess interaction between the degree of OP exposure and PON1 status in terms of risk of BuChE inhibition.

## Figures and Tables

**Table 1:** Characteristics of participants (*n* = 163).^a^

Characteristic	No. (%)
Sex	
Male	163 (100.0)
Missing	0
Race/ethnicity	
Hispanic/Latino	139 (99.3)
Non-Hispanic white	1 (0.7)
Missing	23
Age (years)	
18–24	27 (17.0)
25–34	69 (43.4)
35–49	52 (32.7)
≥ 50	11 (6.9)
Missing	4
Level of education	
Did not attend school	5 (3.6)
Did not complete primary school	16 (11.4)
Primary school	51 (36.2)
Middle school	54 (38.3)
High school	15 (10.6)
Missing	22
Able to read	
In Spanish	139 (98.9)
In English	45 (31.9)
Missing	22
Years employed as a pesticide handler	
≤ 1	21 (19.3)
2–3	33 (30.3)
4–5	20 (18.4)
6–10	21 (19.3)
> 10	14 (12.8)
Missing	54
Location of home	
In town	68 (48.6)
Rural area, away from orchards	21 (15.0)
Rural area, near orchards	20 (14.3)
In/next to orchards	27 (19.3)
Other	4 (2.9)
Missing	23
Reported OP/CB use^b^	
Chlorpyrifos	88 (66.7)
Carbaryl	39 (29.6)
Azinphos-methyl	19 (14.4)
Dimethoate	11 (8.3)
Malathion	6 (4.6)
Other OP/CB	16 (11.2)
Missing	31
Year of participation in study	
2006	89 (54.6)
2007	74 (45.4)
Missing	0
Time since baseline ChE test (days)	
≤ 30	10 (6.2)
31–60	115 (71.0)
61–90	18 (11.1)
> 90	19 (11.7)
Missing	1
Days since last exposure	
Today/yesterday	18 (13.7)
2–7 days ago	66 (50.4)
> 7 days ago	47 (35.9)
Missing	32
Survey language	
Spanish	148 (97.4)
English	4 (2.6)
Missing	11

a Missing values were excluded from percentages. Responses were missing if the participant did not complete the survey, did not know, or refused to answer.

b Participants could select multiple responses.

**Table 2:** BuChE inhibition (mean ± SD) after stratification by *PON1* Q192R genotype and level of plasma PON1 activity (*n* = 163).^a^

	Level of plasma PON1 activity^b^			
Q192R genotype	High	Moderate	Low	*p*-Value^c^
RR	*n* = 12	*n* = 14	*n* = 24	*p* = 0.008
	0.53 ± 6.90%	−0.11 ± 9.42%	−8.22 ± 12.66%	
	Reference	*p* = 0.841	*p* = 0.008	

QR	*n* = 29	*n* = 28	*n* = 24	*p* = 0.069
	−2.06 ± 8.31%	−6.17 ± 9.67%	−7.58 ± 13.24%	
	*p* = 0.302	*p* = 0.014	*p* = 0.017	

QQ	*n* = 13	*n* = 12	*n* = 7	*p* = 0.727
	−9.47 ± 10.88%	−7.23 ± 11.67%	−12.15 ± 11.99%	
	*p* = 0.006	*p* = 0.046	*p* = 0.008	

a *p*-Values were determined based on linear regression with robust standard error estimates.

b Level of plasma PON1 activity is based on AREase activity: high = > 145 U/mL; moderate = 124–145 U/mL; low = < 124 U/mL.

c *p*-Value for trend by tertiles of plasma PON1 activity within each genotype.

**Table 3:** BuChE inhibition in relation to *PON1* Q192R genotype and level of plasma PON1 activity.

		Unadjusted^a^		Adjusted^b^	
PON1 status	No.	β-Coefficient	*p*-Value	β-Coefficient	*p*-Value
Q192R genotype	110		0.062^c^		0.028^c^
RR	33	Reference	—	Reference	—
QR	55	−1.82	0.445	−3.36	0.130
QQ	22	−5.72	0.056	−6.58	0.030
Level of plasma PON1 activity^d^	110	0.061	0.048	0.061	0.053

a Q192R genotype and level of plasma PON1 activity analyzed in separate models using linear regression with robust standard error estimates. This analysis was restricted to 110 records with nonmissing data for all covariates that were included in the adjusted model.

b Q192R genotype and level plasma PON1 activity in same model, and adjusted for year of participation, days since baseline ChE test, age in years, and cumulative exposure score. Multiple linear regression with robust standard error estimates was used.

c Test for trend by number of Q alleles.

d Based on AREase activity (U/mL). The coefficient represents the difference in BuChE inhibition for a one-unit increase in AREase activity.

**Table 4:** Risk of > 20% BuChE inhibition by *PON1* Q192R genotype and level of plasma PON1 activity after adjustment for covariates^a^ (*n* = 110).

PON1 status	Cases (%)^b^	Odds ratio	95% CI	*p*-Value
Q192R genotype				0.033^c^
RR	3 (9)	Reference	—	—
QR	7 (13)	4.02	0.62–26.16	0.146
QQ	5 (23)	10.55	1.17–95.08	0.036
Level of plasma PON1 activity^d^				0.042^e^
High	3 (8)	Reference	—	—
Moderate	4 (12)	1.48	0.17–13.28	0.726
Low	8 (22)	3.01	0.43–21.30	0.269

a Based on unconditional logistic regression with Q192R genotype and tertiles of plasma PON1 activity in the same model, adjusted for year of participation (2006 (2007), days since baseline ChE test (≤ 60, 61–90, > 90), age (18–24, 25–34, 35–49, ≥ 50 years), and cumulative OP/CB exposure score (<−0.85, −0.85 to 0.45, > 0.45).

b Cases defined as > 20% BuChE inhibition from baseline levels. Percentages refer to the proportion of cases within each stratum.

c Test for trend by number of Q alleles.

d Based on AREase activity high = > 145 U/mL; moderate = 124–145 U/mL; low = < 124 U/mL.

e Test for trend by AREase activity modeled as a continuous variable.
